# Ultraviolet/blue light-emitting diodes based on single horizontal ZnO microrod/GaN heterojunction

**DOI:** 10.1186/1556-276X-9-446

**Published:** 2014-08-28

**Authors:** Chia-Fong Du, Chen-Hui Lee, Chao-Tsung Cheng, Kai-Hsiang Lin, Jin-Kong Sheu, Hsu-Cheng Hsu

**Affiliations:** 1 Department of Photonics, National Cheng Kung University, Tainan 70101, Taiwan; 2 Advanced Optoelectronic Technology Center, National Cheng Kung University, Tainan 70101, Taiwan

**Keywords:** ZnO, Microrod, Electroluminescence

## Abstract

We report electroluminescence (EL) from single horizontal ZnO microrod (MR) and p-GaN heterojunction light-emitting diodes under forward and reverse bias. EL spectra were composed of two blue emissions centered at 431 and 490 nm under forward biases, but were dominated by a ultraviolet (UV) emission located at 380 nm from n-ZnO MR under high reverse biases. Light-output-current characteristic of the UV emission reveals that the rate of radiative recombination is faster than that of the nonradiative recombination. Highly efficient ZnO excitonic recombination at reverse bias is caused by electrons tunneling from deep-level states near the n-ZnO/p-GaN interface to the conduction band in n-ZnO.

## Background

ZnO is one of the most potentially useful materials for near-ultraviolet photonic devices such as light-emitting diodes (LEDs) due to its direct wide bandgap energy of 3.37 eV and large exciton binding energy of 60 meV at room temperature (RT) [1-3]. Although ZnO p-n junction LEDs with low luminescence efficiency have recently been reported, [4] ZnO-based LEDs still suffer from difficulty in producing reliable and high-quality p-type doping materials [5-7]. Therefore, the n-ZnO and p-GaN heterojuction devices is suggested as an alternative approach due to their similar lattice structure (wurtzite) and electronic properties [8,9]. Micro/nanostructure LEDs with good crystalline quality and superb waveguide properties are expected to provide an effective route for improving internal quantum efficiency as well as extraction efficiency [10]. To date, various one-dimensional heterojuction micro/nanodevices have been fabricated [11]. Among these structures, the heterojunction LEDs use vertically aligned one-dimensional ZnO structures such as microrods (MRs) and nanorods (NRs) which exhibit better electroluminescence (EL) performance than ZnO film LEDs because the carrier injection efficiency can be enhanced and structural defects are decreased in these micro/nanostructures [12-19]. Few studies have been reported concerning the EL from horizontal ZnO MRs/NRs [10,20-22]. The UV electroluminescence centered around 390 nm in wavelength based on the single ZnO MR/p-GaN [20] and multiple ZnO MRs/p-GaN [21] heterojunction were realized under the forward injection current. In particular, the UV whispering-gallery-mode lasing in an individual ZnO MR-based diode has been demonstrated [10]. A saturated blue emission around 460 nm caused by the interfacial radiative recombination in single ZnO MR/p-GaN at high forward bias was examined [22]. Although those groups have produced the horizontal ZnO MR-based LEDs, a detailed investigation on the origins of the recombination processes is urgently needed for lighting applications. Here, we report one-dimensional hexagonal ZnO MR-based LEDs by simply transferring an individual ZnO MR onto p-type GaN thin film. Two obvious emission bands centered at 431 and 490 nm were obtained under both forward and reverse bias. The EL spectra were dominated by an intense UV emission band under higher reverse bias by reason of the tunneling electrons from GaN assisted by the deep-level states near the n-ZnO/p-GaN interface to the conduction band in n-ZnO. The origins of the distinct electron–hole recombination processes are discussed. Furthermore, the output light-current characteristic was determined to evaluate the high-efficiency electroluminescence performance of the diode.

## Methods

The ZnO MRs were grown on Si (100) substrates by a high-temperature thermal evaporation process. A mixture of ZnO and graphite powders (1:1 in weight ratio) was loaded in an alumina boat serving as the source material. The boat was centered inside a 2.5-cm quartz tube in a tube furnace. A clean Si substrate was placed on top of the Al_2_O_3_ boat to collect samples. The furnace was heated to 1,050°C at a rate of 20°C/min and kept at that temperature for 60 min. After the furnace had naturally cooled down to room temperature, the ZnO MRs were deposited on the Si substrate. To construct the LED, a p-type GaN layer was grown on a (0001) sapphire substrate with hole concentration and mobility of 10^17^ cm^−3^ and 10 cm^2^/V-s, respectively, was used as the hole injection layer. A thin layer of PMMA was partly coated on the p-type GaN film to serve as an insulating layer. After the substrate was heated at 50°C for 20 min to improve the quality of the PMMA, a single ZnO MR was transferred to the prepared p-GaN substrate and crossed the boundary with the p-GaN and PMMA. Finally, the ZnO MR was fixed by Ag paste which served as the cathode, while another Ag electrode on the GaN film worked as the anode. The sample morphology was examined with a high-resolution Zeiss FEG scanning electron microscope (SUPRA 55, Carl Zeiss, Oberkochen, Germany). The polarized micro-Raman spectra of the individual ZnO MR were measured using a Horiba Jobin-Yvon iHR320 spectrometer (Horiba, Kyoto, Japan) in a backscattering configuration. The 532-nm line of a frequency-doubled Nd:YAG laser with 4.2-mW power was used for off-resonance excitation. The I-V measurements were carried out with a Keithley 2400 source meter (Cleveland, OH, USA). Micro-photoluminescence (μ-PL) and EL measurements were conducted by the above spectrometer. The optical source was provided by a 0.3-mW He-Cd laser with the wavelength of 325 nm. All measurements were performed at room temperature.

## Results and discussion

**Figure 1:**
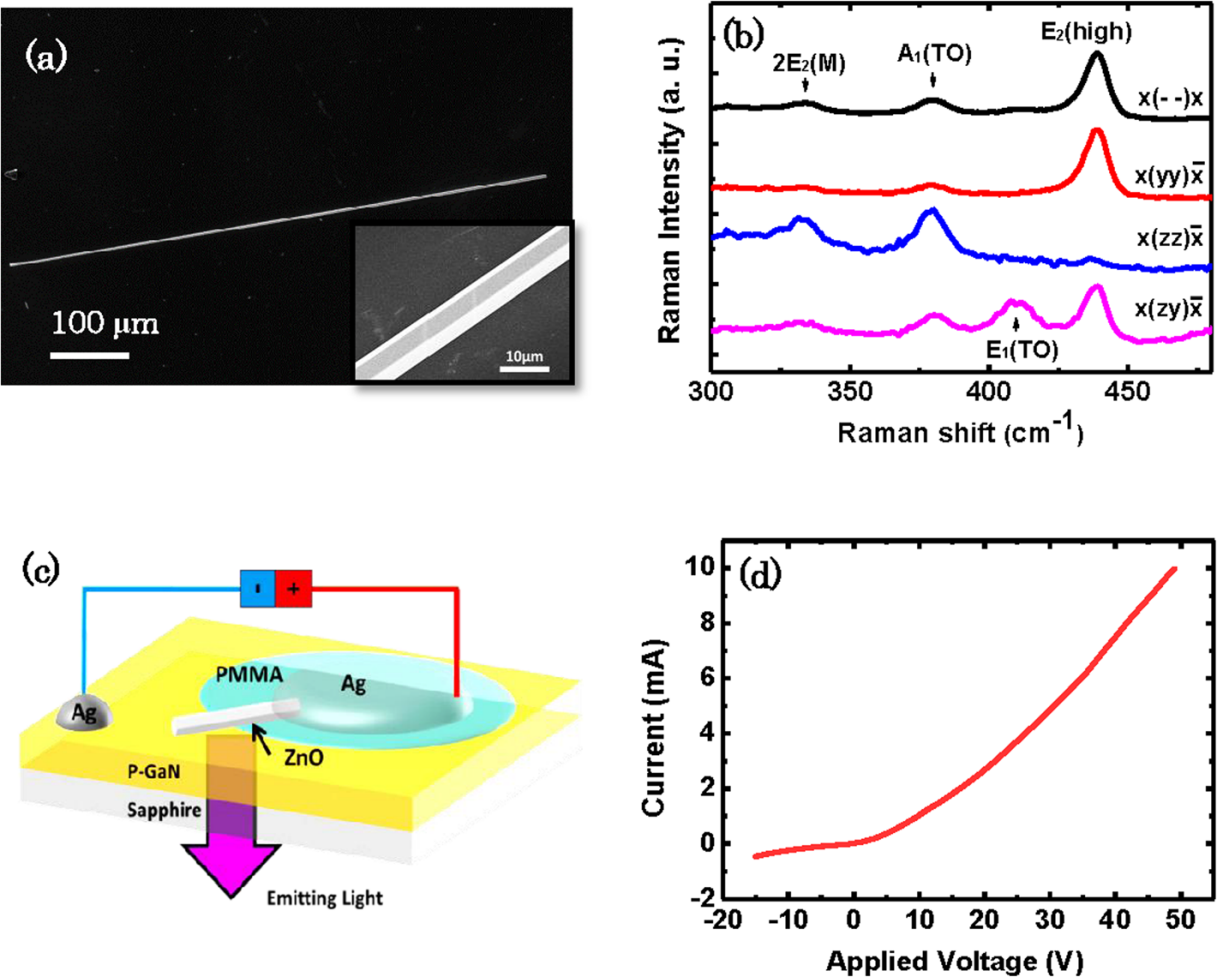
**SEM image**, **polarized μ-Raman spectra, schematic, and I-V characteristics. (a)** SEM image of an individual ZnO MR. The inset shows the enlarged SEM image. **(b)** Polarized μ-Raman spectra of the ZnO MR. **(c)** Schematic of a light emitting diode device. **(d)** The I-V characteristics of the heterojunction device.

Figure 1a shows uniform size of 700 μm in length of the individual ZnO microrod. The inset of the SEM image in Figure 1b reveals that the MR has a hexagonal cross-section and smooth side facets that are 6 μm in diameter. The upper trace of the Figure 1a shows the polarized Raman spectra results. Three distinct peaks at 380, 410, and 437 cm ^−1^ were observed, which can be identified to A_1_(TO), E_1_ (TO), and E_2_ (high) modes, respectively. The peak at 331 cm^−1^ can be assigned to the second-order Raman scattering arising from zone-boundary phonons 2-E_2_(M) of ZnO. A strong A_1_ (TO) mode in the parallel polarization configuration and a predominant E_2_ (high) mode in the perpendicular polarization configuration indicate that the MR has a c-axis single crystalline wurtzite structure [23,24]. The schematic diagram of the n-ZnO MR/p-GaN heterostructure LED is shown in Figure 1c. Figure 1d displays a current–voltage (I-V) curve for the device and presents a typical rectifying curve of the heterostructured diode device, suggesting the formation p-n junctions at the interface. The reverse turn-on voltage is 6 V.

**Figure 2:**
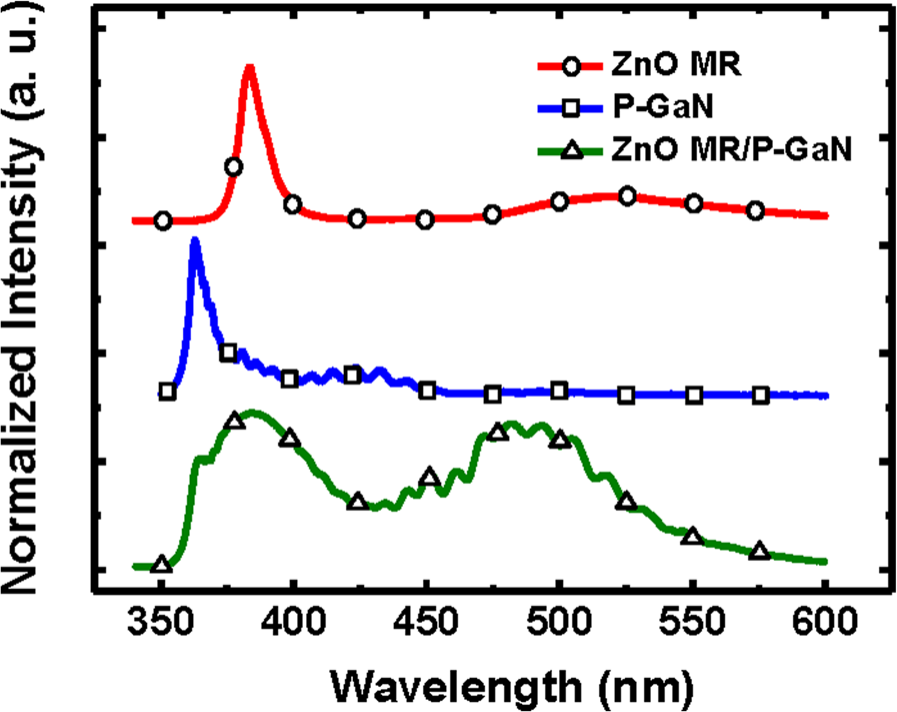
The room-temperature μ-PL spectra of single ZnO MR, p-GaN substrate, and ZnO/p-GaN heterojunction.

**Figure 3:**
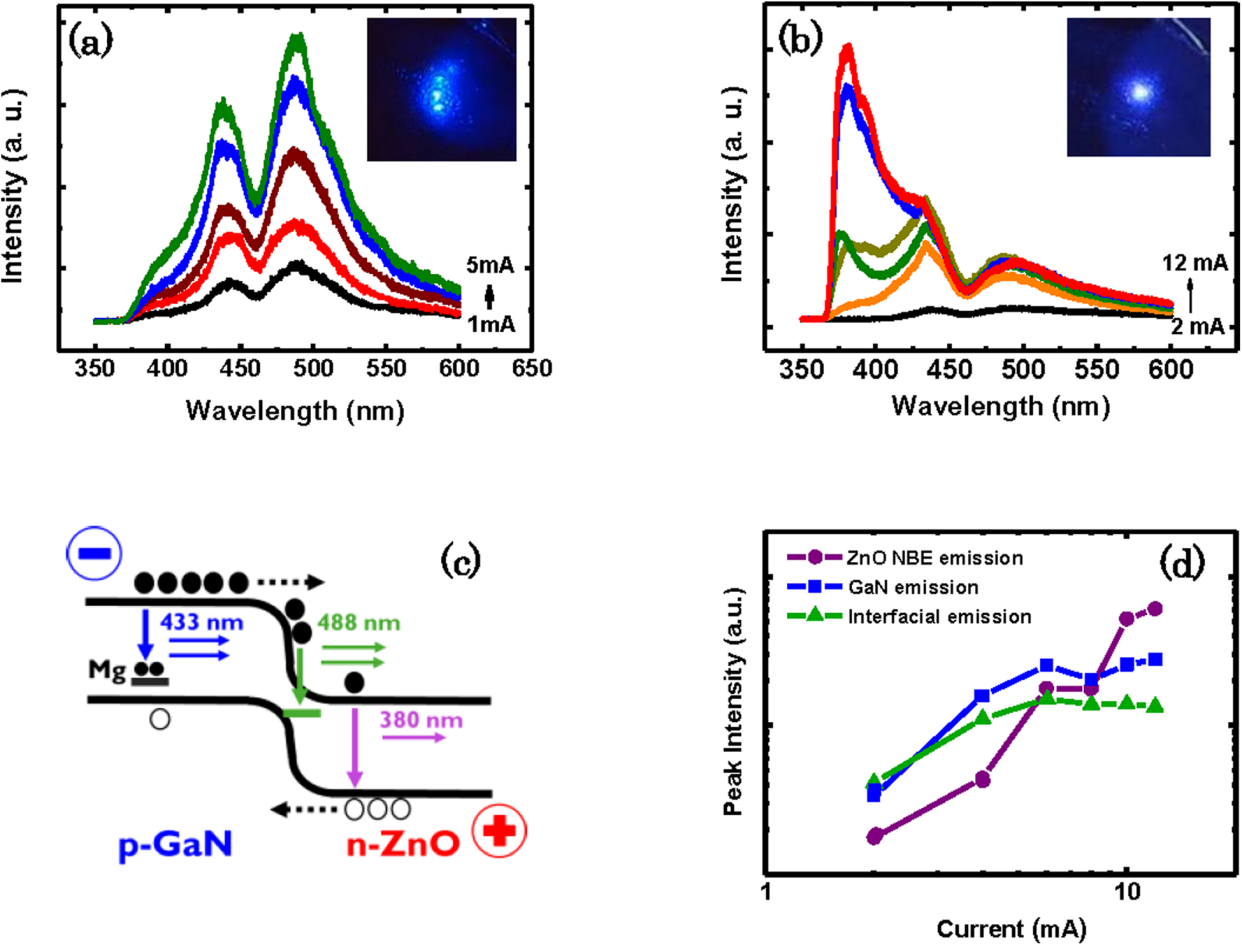
**The room temperature EL spectra of n-ZnO/p-GaN heterojunction LED (a) under various forward biases and (b) under reverse biases.** The lighting images under the biases (+36 V and −30 V) are shown in the insets of (a) and (b), respectively. **(c)** The band diagram of the n-ZnO/p-GaN heterojunction devices under reverse bias. **(d)** The three light output intensities of the heterostructure as a function of injection current under reverse bias.

Figure 2 shows the PL spectra of the single ZnO microrod, p-GaN films, and ZnO/GaN heterostructure measured at room temperature. The PL spectrum of the ZnO microrod consists of an intense near-band-edge (NBE) UV emission centered at 380 nm attributed to the radiative recombination of free excitons and a broad green band due to the defect emission related to oxygen vacancies or zinc interstitials [25]. The p-GaN film exhibits the NBE-related UV emission peak at around 362 nm and the broad blue emission peak centered at 445 nm which can be attributed to transitions from the conduction band or shallow donors to deep Mg acceptor levels [26]. The appearance of several oscillations is due to the interference effects of the thickness of the smooth GaN film. The bottom line in Figure 2 shows the PL result of the ZnO/GaN heterostructure. The pumping laser beam can penetrate through the ZnO microrod into the underlying p-GaN. One additional emission peak centered around 490 nm could be obtained, which is attributed to the emissions arising from the carrier recombination in regions near the heterojunction interfaces [27].The EL device can be operated at both forward and reverse bias current. The EL spectra of the heterojunctions under various forward biases are shown in Figure 3a. Under high forward bias current, there are two dominant emissions centered at 430 and 490 nm and a relatively weak emission of 380 nm at the short-wavelength shoulder of the first emission peak. The origin of the EL emission of heterojunction diodes can be confirmed by comparing the EL with PL spectra. The emission around 430 nm is ascribed to the Mg acceptor levels in the p-GaN thin film. The blue emission around 490 nm comes from the ZnO MR/p-GaN interface; the electron would be captured by the deep-level states near the interface. The UV emission band around 380 nm is attributed to the excitonic emission in ZnO MR. Consequently, with the increase of the bias, a UV emission at 380 nm can be observed, but the EL spectra are still dominated by the blue emission.

More importantly, the excitonic emission of ZnO MR dramatically increases and becomes a distinct peak as the applied reversed biases increase as shown in Figure 3b. The EL spectra are dominated by the p-GaN emission under forward biases, whereas they are dominated by the n-ZnO emission under reverse biases. The inset in Figure 3a,b shows the EL image of the LED under the biases in a dark room, emitting bright blue and white light, respectively. Note that they are visible to the naked eye. The mechanism of carrier recombination of EL can be interpreted by the energy band diagram as shown in Figure 3c. Figure 3d displays the intensity of the three emission peaks as a function of the reverse bias. Under low reverse bias current, due to the lower mobility in the p-GaN, all of the radiative recombination mainly occurs in the p-GaN and interfacial layer. When the reverse bias current increases, the radiative recombination occurs in three places - the p-GaN, interfacial layer, and ZnO MR. Until the applied current exceeds a certain value, the carrier recombination in the p-GaN no longer increases because of the limited hole concentration in the p-GaN thin film. Finally, the excitonic emission of ZnO MR dramatically increases and becomes a distinct peak as the applied reversed bias current increases. The three peak intensities of the ZnO emission under reverse bias are depicted as a function of injection current in a log-log scale. Using the formula *I*_em_ ~ *I*^m^, where *I*_em_ is the peak intensity, *I* is the injection current, *m* is an index, the dependence curve can be fitted, and the fitting results reveal that the device shows a superlinear relationship with *m* = 2. This implies that, compared to the reported heterojunction device [28], the effect of defect-related nonradiative recombination is negligible and almost every injected carrier leads to the emission of a photon under reverse bias. In contrast, the emissions from GaN and interfacial recombination both show superlinear dependence under low current injection; however, the luminescence peak intensities increase sublinearly at higher injected currents (*I* > 7 mA). This indicates that nonradiative recombination is responsible for the output saturation.

**Figure 4:**
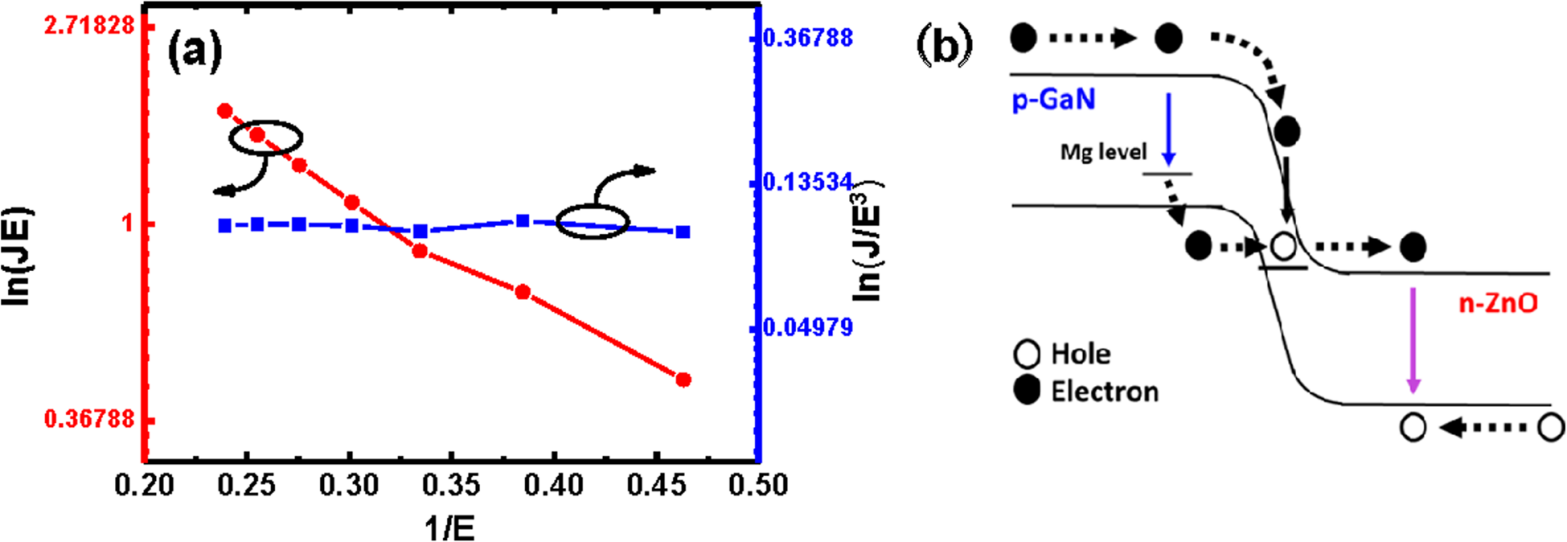
**The linear dependence and the carrier transports and recombination mechanisms. (a)** Plots of ln(*J* · *F*) versus *E*^−1^and ln(*J*/*E*^3^) versus *E*^−1^of the n-ZnO/p-GaN heterojunction LED at reverse breakdown bias. **(b)** The band diagram of the p-GaN/n-ZnO heterojunction under the reverse breakdown bias.

To understand the carrier transport mechanisms based on the electron from the band-to-band tunneling or deep-level states to the conduction band of n-type ZnO at reverse breakdown bias, we examined the electrical properties of the device in detail. The tunneling current density *J* from a deep-level state to a continuum of free states in a conduction band can be expressed as follows [9,29]:

(1) $$J\propto P=\frac{A}{E}\exp\left(-\frac{B}{E}\right)$$

where *P* is the tunneling ionization rate, *E* is electric field, and *A* and *B* are constants. On the other hand, the band-to-band tunneling from the occupied valence band states directly to the empty conduction band states at reverse breakdown bias is given by [30]:

(2) $$J=CE^{3}\exp\left(-\frac{B}{E}\right)$$

where *C* and *D* are constants. Using Equations 1 and 2, ln (*J* · *E*) versus *F*^−1^ and ln (*J*/*E*^3^) versus *E*^−1^ plots can be plotted by the studied I–V characteristics of the LED at reverse breakdown as shown in Figure 4a. The linear dependence between ln (*J* · E) and *E*^−1^ indicates that the reverse breakdown is dominated by the electron tunneling from the deep-level states near the n-ZnO/p-GaN interface to the conduction band in n-ZnO. In contrast, the constant of the ln (*J*/*E*^3^) versus *E*^−1^ plot indicates that the contribution of the electron tunneling from the valence band in p-GaN directly to the conduction band in n-ZnO is much weaker. This finding may be a result of the narrower energy barrier width for electron tunneling from the valence band in p-GaN than that from the deep-level states near the n-ZnO/p-GaN interface. We summarize the band diagram of the n-ZnO MR/p-GaN heterojunction under the reverse breakdown bias to illustrate the carrier transports and recombination mechanisms in Figure 4b.

**Figure 5:**
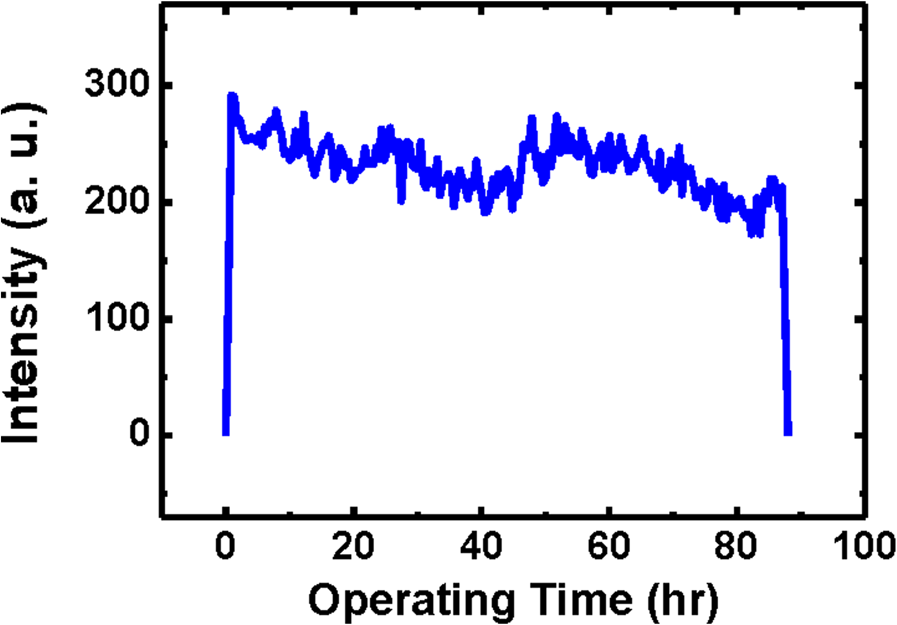
EL emission intensities as a function of time.

To assess the suitability of the studied diode to practical LED applications, a preliminary stability study of EL performance was conducted. Figure 5 displays the EL intensities of the device working under reverse bias of 40 V. The EL intensities did not decrease significantly even after over 80 h of operation. To date, there is no literature demonstrating the stability of an individual horizontal ZnO MR/p-GaN heterojunction. The stability of the diode was comparable to other devices based on the vertical n-ZnO NWs/p-GaN structure [17,31]. This measurement proves that this EL device displays good stability and reproducibility.

## Conclusions

In summary, we have obtained UV and blue dual-color LED based on single ZnO MR and p-GaN heterojunction under forward and reverse biases, respectively. The origin of the EL emission was confirmed by comparing the EL and PL spectra. For the excitonic ZnO emission, the rate of radiative recombination is faster than that of the nonradiative recombination under reverse bias. The tunneling electrons assisted by the deep-level states near the n-ZnO/p-GaN interface to the conduction band in n-ZnO result in the efficient ZnO excitonic luminescence under reverse bias. This stable UV/violet EL device should have potential applications in many areas, including multicolor lighting, displays, and lighting decoration.

## Competing interests

The authors declare that they have no competing interests.

## Authors’ contributions

CFD and CHL carried out the characterizations of the device and PL measurements and participated in data interpretation. CTC performed the Raman spectra measurement. KHL synthesized the ZnO microstructures. JKS provided the GaN thin films and participated in data interpretation. HCH initiated the study, designed all the experiments, and analyzed the data. CFD and HCH wrote the manuscript. All authors read and approved the final version of the manuscript.
